# Deep Learning Based Syndrome Diagnosis of Chronic Gastritis

**DOI:** 10.1155/2014/938350

**Published:** 2014-03-05

**Authors:** Guo-Ping Liu, Jian-Jun Yan, Yi-Qin Wang, Wu Zheng, Tao Zhong, Xiong Lu, Peng Qian

**Affiliations:** ^1^Basic Medical College, Shanghai University of Traditional Chinese Medicine, Shanghai 201203, China; ^2^Center for Mechatronics Engineering, East China University of Science and Technology, Shanghai 200237, China; ^3^Technologies and Experiment Center, Shanghai University of Traditional Chinese Medicine, Shanghai 201203, China

## Abstract

In Traditional Chinese Medicine (TCM), most of the algorithms used to solve problems of syndrome diagnosis are superficial structure algorithms and not considering the cognitive perspective from the brain. However, in clinical practice, there is complex and nonlinear relationship between symptoms (signs) and syndrome. So we employed deep leaning and multilabel learning to construct the syndrome diagnostic model for chronic gastritis (CG) in TCM. The results showed that deep learning could improve the accuracy of syndrome recognition. Moreover, the studies will provide a reference for constructing syndrome diagnostic models and guide clinical practice.

## 1. Introduction

In recent years, the standardization and objectification of TCM diagnosis have gradually became a research hotspot with the development of mathematical statistics, data mining, and pattern recognition technology. Many researches are emerged in large numbers. An entropy-based partition method for complex systems is applied to establish endothelial dysfunction diagnostic criteria for Yin deficiency syndrome. Moreover, the experimental results are highly consistent with the findings of clinical diagnosis [1]. Su et al. [2] employed the correlation coefficient, similarity D, the angle cosine, and spectral similarity to study the correlation between the symptoms (signs) and the five syndromes of liver cirrhosis. The research can provide a basis for differentiating patients with nonspecific clinical manifestations. Multilabel learning [3] combined with the feature selection had been used to improve the syndrome recognition rate of chronic gastritis.

Although a large number of machine learning methods have been used in the standardization and objectification of TCM diagnosis, researchers can provide a reference for clinical syndrome differentiation. However, in clinical practice, diagnosis of TCM is from the brain and has some hierarchical nature, complexity, and nonlinearity. There is a complex and nonlinear relationship between symptoms (signs) and syndrome. Most of the algorithms are not considering the hierarchical nature of diagnosis from the brain's cognitive perspective. This is likely to cause misunderstanding and bias.

Inspired by the hierarchical structure of the brain, neural network researchers have been working on multilayer neural network. Back propagation algorithm (BP) is a classical multilayer network algorithm, but the theoretical and experimental results showed that BP was not suitable for training the data with multiple hidden layer units [4]. Traditional machine learning and signal processing techniques were only to explore the shallow structure containing a single layer and nonlinear transformation. Typical shallow layer learning included traditional hidden Markov model (HMM), conditional random fields (CRF), maximum entropy model (MaxEnt), and support vector machine (SVM). The function ability of representing shallow layer structure has its limitations. However, deep learning [5] can succinctly represent complex functions.

Hinton Research Group proposed the deep network and deep learning concept in 2006. Hinton et al. [6, 7] proposed unsupervised training drill greedy algorithm for solving optimization problems and then proposed the automatic multiencoder deep belief networks based on the deep structure (DBN). LeCun et al. [8] proposed convolutional neural networks (CNNs), the first true multilayer structure learning algorithms, which use relative spatial relationships, reducing the number of parameters to improve the performance of BP training. In addition, the study of deep learning also appeared in many deformed structures such as automatic denoising encoder [9, 10], DCN [11] and sum-product [12]. Deep learning method has been applied to machine vision [13–15], speech recognition [16, 17], and other areas to improve data classification and identification of effects and set off a new craze machine field.

Deep learning is distinctly more in line with the human brain thinking; it can use high-dimensional abstract features to express some of the original low-dimensional features. It is a good method to find the relationship between the symptoms each other and between the symptoms and syndromes. This idea is consistent with the diagnosis ideas of TCM.

At the same time, patients may simultaneously have more than one syndrome in clinical practice. Therefore, in this paper, we proposed to apply the deep learning method to establish the multilabel learning model of CG. Through the deep learning algorithm, we try to find a complex and nonlinear relationship between symptoms and syndromes of CG and to improve the syndrome cognition rate of CG.

## 2. Material and Methods

### 2.1. Research Subjects

Chronic gastritis (CG) samples were collected from a clinic, inpatient department, and gastroscopy room of the digestive system department of the Longhua Hospital, the Shuguang Hospital of Shanghai University of Traditional Chinese Medicine, the Xinhua Hospital, the Putuo District Central Hospital, and the Shanghai Hospital of Traditional Chinese Medicine. The Shanghai Society of Medical Ethics approved this work. All patients signed an informed consent form. A total of 919 valid subjects were enrolled after excluding cases with TCM inquiry diagnosis scales that lacked information or cannot be diagnosed with CG. Among the 919 patients, 354 were male (38.5%, with an average age of 44.61 yr ± 14.54 yr) and 565 were female (61.5%, with an average age of 48.70 yr ± 12.74 yr).

### 2.2. Inclusion Criteria

Patients who met the diagnostic standards for CG and TCM syndromes and patients who were informed and have agreed to join this investigation were included.

### 2.3. Diagnostic Criteria

Western Diagnostic Standards include diagnosing whether a patient has CG based on gastroscopy results, pathologic results, and clinical performance, according to the Consensus of National Seminar on CG held by the Chinese Medical Association Digestive Diseases Branch in 2007 [18].

Chinese Diagnostic Standards include the following eight syndromes (patterns):

 (1) damp heat accumulating in the spleen-stomach;
 (2) dampness obstructing the spleen-stomach;
 (3) spleen-stomach qi deficiency;
 (4) spleen-stomach cold deficiency;
 (5) liver stagnation;
 (6) stagnated heat in the liver-stomach;
 (7) stomach Yin deficiency;
 (8) blood stasis in the stomach collateral.

We referred to the diagnoses in “Guideline for Clinical Research of New Traditional Chinese Medicine” [19] issued by the Ministry of Health and “National Standard of People's Republic of China: Syndrome Part of TCM Clinical diagnosis and Treatment Terminology” [20] issued by the China State Bureau of Technical Supervision.

### 2.4. Exclusion Criteria

1. mentally ill patients and patients with other severe systemic diseases;
2. patients who have difficulty in describing their conditions;
3. patients who are not informed or refuse to cooperate.

### 2.5. Method for Establishing TCM Inquiry Diagnosis Scales

The research group was composed of Shanghai senior clinical experts on the digestive system, clinical doctors, and researchers. The final TCM inquiry diagnosis scales were drafted based on past experience in the production of scales [21], a wide range of literature about TCM spleen and stomach diseases, related documents in core magazines and journals for over 15 years, and reports about the frequency of symptoms associated with syndromes in CG diseases in TCM. The scales were also amended and fixed by two rounds of expert consultation and statistical tests. The scales include eight dimensions such as cold or heat, sweat, head, chest and abdomen, urine and stool, diet and taste, sleep, mood, woman aspects, and contents of disease history, inspection, and palpation. More than 113 variables were ultimately included in these scales.

### 2.6. Investigation Methods

The clear definitions of symptoms, the specific methods, and the order of inquiry diagnosis were given in the scales. All samplers must have undergone unified training. The group members assemble regularly and discuss the information of typical patients to ensure the consistency of the collected data.

### 2.7. Diagnosis Methods

Three senior chief doctors with plenty of experience in clinical practices were invited for inquiry diagnosis of the cases in terms of the CG diagnostic standards made by our research group. If two of them have the same diagnosis results, the case was included. Otherwise, the case was not adopted until at least two of them came to the same conclusion.

### 2.8. Data Input and Process Methods

1. Build a database with Epidata software.
2. Input data two times independently.
3. The Epidata software compares the two data sets and checks out mistakes.
4. Check the investigation form logically in case of filling errors.

### 2.9. Analysis Methods

#### 2.9.1. Multilabel Learning Based on Deep Learning

Deep belief network (DBN) is a deep architecture, which is suitable to deliver nonlinear and complicated machine learning information. At the same time, the process of syndrome differentiation is considered to be nonlinear and complicated. Applying the DBN based multilabel on syndrome differentiation modeling is more appropriate. A DBN model is actually a multilayer perception neural network with one input layer, one output layer, and several middle hidden layers unit. The higher-level layer connects to its lower layer by a Restricted Boltzmann Machine (RBM) which uses the result of the lower layer to activate the next higher-level layer.

**Figure 1:**
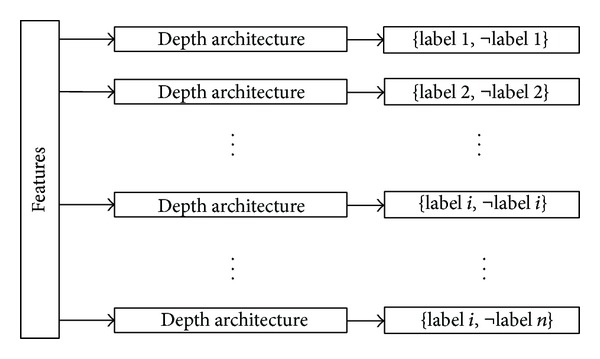
The process of deep learning multilabel learning.

Our study applies the common deep learning method to deal with multilabel learning problem. The multilabel classification algorithms can be generally divided into two different categories [22]: problem transformation methods and algorithm adaptation methods. Some of them consider the correlations among the labels and some of them do not. For the convenient reason, we chose a simple method that ignores the correlations among labels to build the model, that is, binary relevance (BR) method. The deep learning model deep belief network (DBN) will be combining with binary relevance method, respectively, to deal with multilabel learning task. Binary relevance (BR) approach [23] directly transforms multilabel problem *N* binary classifiers Hn: X → {l, −l}, and each independent classifier deals with only one label. In this paper, DBN will take the place of the *N* binary classifiers. For example, multilabel learning model of CG syndrome diagnosis will be established for accomplishing six labels with six deep learning processes in this paper. The details of the process of multilabel learning methods based on deep learning are shown in Figure 1.

#### 2.9.2. Multilabel Learning Framework Based on Deep Belief Nets

**Figure 2:**
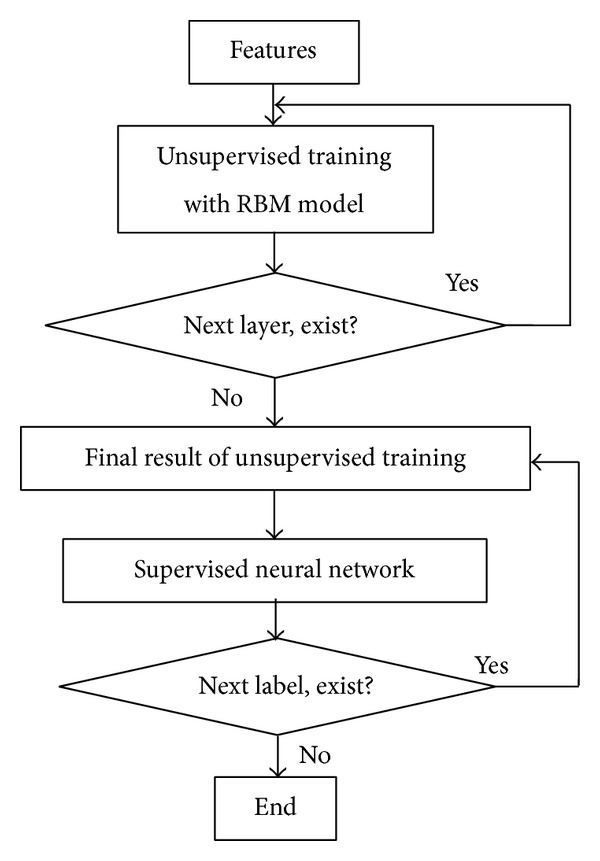
The process of deep belief nets multilabel learning.

The following text will describe the learning process of deep belief network in detail. In this model, the original features were used directly in the multilabel learning of deep belief network.  Figure 2 shows the approximate learning process of multilabel learning based on deep belief network. We put sample features and relevant parameters into the unsupervised RBM training model for training and then shift up the hidden layer to higher layer. This process is repeatedly executed until current hidden layer becomes the highest hidden layer, and so on; several unsupervised RBM models can be trained from visible layer to highest hidden layer and then obtain an initial set of the weighting parameters. Later on, the samples' original features are taken as the input layer of neural network, a label is chosen as output layer of neural network, and the middle hidden layer is taken as the hidden layer of neural network; a neural network model is trained from visible input layer to output layer. The weighting parameters in every layer can further be updated through the forward propagation and afterward propagation. After training, the category labels of training have been finished. Then, another label is chosen to be trained, until all labels are finished. The predicting process is the same as its training process, which means the labels are also predicted one by one. When each label is predicted, the neural network is used, which takes the samples' features as the input layer of the number of hidden layers and the number of hidden layer units that stayed the same as in training process and executes the prediction through the forward propagation with the weighting parameters in every layer. We can map the corresponding higher expression of original features through trains corresponding model in the lower level to higher until the highest level expression results is presentation. The details of the process of multilabel learning methods based on deep belief nets are shown in Figure 2.

## 3. Experimental Design and Evaluation

The evaluation index of single label learning is usually accuracy, recalling rate and F1 measure value, but evaluation is different from single-label learning. The following five evaluation metrics specifically designed for multilabel learning are expressed as follows [24].

Average precision evaluates the average fraction of labels ranked above a particular label *y* ∈ *Y*, which actually are in *Y*. The performance is perfect when avgprecS(*f*) = 1; the bigger the value of avgprecS(*f*) is, the better the performance is:

(1) avgprecS(f) =1p∑i=1p1|Yi|  ×∑y∈Yi|{y′ ∣ rankf(xi,y′)≤rankf(xi,y),y′∈Yi}|rankf(xi,y).

Coverage evaluates how far on average we need to go down the list of labels to cover all the proper labels of the instance. It is loosely related to precision at the level of perfect recall. The smaller the value of coverageS(*f*) is, the better the performance is:

(2) coverageS(f)=1p∑i=1pmax⁡y∈Yi  rankf(xi,y)−1rankf(xi,y)=1−f(xi,y).

Ranking loss evaluates the average fraction of label pairs that are reversely ordered for the instance. The performance is perfect when rlossS(*f*) = 0; the smaller the value of rlossS(*f*) is, the better the performance is:

(3) rlossS(f)=1p∑i=1p1|Yi||Yi¯||{(y1,y2) ∣ f(xi,y1)≤f(xi,y2),(y1,y2)∈Yi×Yi¯}|,

where $\bar{Y}$ denotes the complementary set of *Y* in *y* · *y* = {1, 2,…, *Q*} being the finite set of labels.

Hamming loss evaluates how many times instance-label pairs are misclassified, that is, a label not belonging to the instance is predicted or a label belonging to the instance is not predicted:

(4) $$\begin{array}{c} \text{hlos}{\text{s}}_{\Gamma }\left(f\right)=\frac{1}{m}\overset{m}{\underset{i=1}{\sum }}\frac{1}{n}\left|f\left(x_{i}\right)\Delta Y_{i}\right|, \end{array}$$

where Δ denotes the symmetric difference between two sets.

One-error evaluates how many times the top-ranked label is not in the set of proper labels of the instance. The performance is perfect when one-error_Γ_(*f*) = 0:

(5) $$\begin{array}{c} \text{one-erro}{\text{r}}_{\Gamma }\left(f\right)=\frac{1}{m}\overset{m}{\underset{i=1}{\sum }}\underset{y\in Y}{\text{argmax}}f\left(x_{i},y\right)f\left(x_{i},y\right)\notin Y_{i}. \end{array}$$

For any predicted *π*, *π* equals 1 if *π* holds and 0 otherwise. Note that, for single-label classification problems, a one-error is identical to an ordinary classification error.

## 4. Results 

We compared the model performance with different nodes' numbers of hidden layer and different multilabel learning algorithms. At the same time, we compared accuracy rates of 6 syndromes using DBN with different hidden layer. The results are shown in the following sections, respectively.

### 4.1. Comparison of Model with Different Nodes' Numbers

In order to illustrate the performance of deep learning framework on chronic gastritis inquiry data, a series of experiments have been carried out. Firstly, to confirm appropriate value of the deep architecture parameter, we set an experiment to confirm the scale of node in each hidden layer. Secondly, deep learning multilabel framework will be compared with other multilabel learning algorithm with either feature select or not. Finally, we compared the accuracy rates in 6 syndromes using different multilabel methods. In the experiments, five evaluation measures are employed: average precision, coverage, hamming loss, one-error, and ranking loss. Average precision expresses “the bigger the better” and the others express “the smaller the better.” The symbol “↓” indicates “the smaller the better” while “↑” indicates “the bigger the better.” Tenfold cross validation is employed on both data sets in order to predict reliably. A symbol “±” connects the means of classification result calculated ten times and their standard deviations. The best results are represented in bold.

Firstly, we experiment on an only one hidden layer DBN to find an appropriate node number value hid in the hidden layer; hid is chosen from [5, 10, 20, 30, 40, 50, 60, 70, 80,   90, 100, 200, 300]. For the process speed, the samples will be handled in batches, each batch containing 100 samples. The other parameters: the learning rate is set to 0.1, the biggest iterations are set to 100, the smooth is set to 0.5, and the damping factor is set to 2e-4.  Table 1 shows the results of five evaluation measures of DBN with one layer. The best results are represented in bold.

As shown in Table 1, when hid = 80, the experimental results in five evaluation standards, as a whole, are the best, where average precision is 0.824, coverage is 0.158, one-error is 0.278, and ranking loss is 0.116 which achieves the best and hamming loss is 0.139 which is worse than the best result (0.135). But when the hid exceeds 30, the results of all the values of hid show little difference, which indicate that as long as there are enough hidden nodes and full learning, the experimental results cannot show too much difference.

### 4.2. Comparison of Model with Different Multilabel Learning

We selected the best result for one hidden layer and its optimal nodes' number DBN model and compared the five evaluation parameters obtained using ML-KNN, Ensembles of Classifier Chains (ECC), BSVM, BP-MLL, Rank-SVM, CLR, REkAL, and LEAD algorithms. For BSVM, we chose the kernel function as linear; for ML-KNN, we set the neighbor number to 10 and chose the Euler distance to measure the sample distance; for Rank-SVM, we set the maximum iterations as 50 and chose the linear kernel function; for BP-MLL, we set the number of hidden neurons layer as 20% of the number of features and set the number of neural node as 100; for CLR and ECC, we set the size of integration as 10 and set the sample proportion as 67%; for REKAL, we set the size of subset as 3 and chose LP as the multiclass algorithm. The results are shown in Table 2.

As shown in Table 2, the result of DBN was significantly better than that of other algorithms. Although DBN is actually a neural network model as well as BP-MLL, the result shows that DBN is obviously superior to BP-MLL with 31.5% higher in average precision measure. It indicates that DBN model is better to deal with TCM CG inquiry data than BP-MLL.

### 4.3. The Comparison of Accuracy Rates of 6 Syndromes

In order to have a further discussion on the effect of the depth of deep architecture, the DBN method was compared with different numbers of layers of accuracy rates for various syndromes. The recognition accuracies of the six common syndromes of CG are shown in Table 3.

As shown in Table 3, there are four syndromes with the DBN algorithm that achieved the highest accuracy rate, that is, the pattern of damp heat accumulation in the spleen-stomach, dampness obstructing the spleen-stomach, spleen-stomach qi deficiency, and liver stagnation achieved at 90.1%, 81.2%, 75.3%, and 83.9%, respectively, followed by BSVM, Rank-SVM, and ML-kNN, whose performances are almost the same. BP-MLL performed second best on the pattern liver stagnation with 83.1% but performed the worst with the other three syndromes. For the pattern of spleen-stomach cold deficiency, the accuracy of DBN has very close performance with ML-kNN and BP-MLL at 96.6%, followed by BSVM at 94.3% and Rank-SVM only at about 80%. For the pattern of stagnated heat in the liver-stomach, BP-MLL algorithm achieved the highest accuracy rate at 91.0%, which is only 0.2% and 0.5% higher than ML-kNN and DBN, and Rank-SVM has the lowest accuracy of 79.9%.

From the comparison of experimental results, DBN method obtains the satisfied comprehensive performance in the multilabel learning for syndrome classification on CG data.

## 5. Discussion

A syndrome is a unique TCM concept. It is an abstractive conception of a variety of symptoms and signs. It is a pathological summarization of a certain stage of a disease and it covers disease location, etiology, and the struggle between the body's resistance and pathogenic factors. Different syndromes have different clinical manifestations. Symptoms, which are the external manifestations of a disease and a syndrome, refer to subjective abnormalities and the abnormal signs of patients elicited by doctors using the four diagnostic methods. The etiology, location, nature, the struggle between the body's resistance and pathogenic factors, and the condition at a certain stage of the disease process are highly summarized using syndrome differentiation. Syndrome differentiation involves three steps: (a) determining symptoms and signs through inspection, auscultation, inquiry, and palpation; (b) making an overall analysis of the information; and (c) making a diagnostic conclusion. All these steps are based on TCM theory.

**Figure 3:**
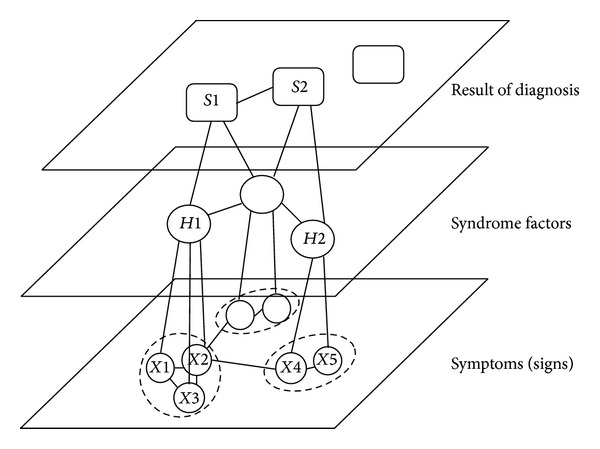
The TCM syndrome diagnosis of the hierarchical structure diagram.

Figure 3 shows the TCM syndrome diagnosis of the hierarchical structure diagram. *X*1, *X*2,… and *X*5 are directly observed and we call them symptoms (signs) variables. In this study, it denotes the symptoms and signs of CG. *H*1 and *H*2 are the syndrome factors, which are the preliminary summarize of syndrome and the foundation of the syndrome diagnosis. *S*1 and *S*2 are the results of the syndrome diagnosis. *H* and *S* are indirectly measured through their manifestations and we call them latent variables, which represent the different hierarchical syndromes of CG.

In clinical syndrome diagnosis, there is certain complex and nonlinear relationship between symptoms and each other and between symptoms and syndromes. The occurrence of a symptom may be accompanied by other symptoms together.

Multiple symptoms concurrent phenomena can be understood: some abstract syndromes factors can represent the collection of several concurrent symptoms. This syndrome diagnosis hierarchy is consistent with the human brain cognitive.

In the traditional information methods of TCM, most research is not considered from a cognitive point of view and from the TCM nonlinear, complex, and multilayered aspects. Simple feature selection is likely to cause the incomplete expression of feature subset and feature conversions easily lead to uncertainty.

Deep learning can use high-dimensional abstract features to express some of the original low-dimensional features without the need for the person to participate in the selection of features. Therefore, deep learning is more consistent with human brain's cognitive thinking. This idea is consistent with the diagnosis ideas of TCM. It is a good method to find the relationship between the symptoms and each other and between the symptoms and syndromes. This idea is consistent with the diagnosis ideas of TCM.

This paper introduces the basic concept of the deep learning method, using the DBN to establish a multilabel learning algorithm and apply this established algorithm on the TCM syndrome differentiation of CG. Firstly, a simple RBM model of different numbers of hidden layer node was tried to find out the appropriate layer on CG. The best result is when the scale of nodes is 80. The average precision, coverage, one-error, and ranking loss were the best; they were 0.823, 0.158, 0.278, and 0.116. Only the hamming loss gets an old stuff value of 0.139 and then the multilabel learning based on DBN was compared with other popular multilabel learning algorithms on CG data in both multilabel learning task and single label learning task. In the multilabel learning task, the multilabel learning based on DBN achieves the best in all the five evaluation measures, especially the average precision (82.3%) being 2% higher than LEAD which is the second best performance with 80.3%. In the single label-learning task, each syndrome was treated as single label classification by various algorithms: ML-kNN, BSVM, BP-MLL, Rank-SVM, and DBN. DBN achieves better than other algorithms with five syndromes, that is, the pattern of damp heat accumulation in the spleen-stomach with the accuracy 90.1%, dampness obstructing the spleen-stomach with the accuracy 81.2%, spleen-stomach qi deficiency with the accuracy 75.3%, spleen-stomach deficiency cold with the accuracy 96.6, and liver stagnation with the accuracy 83.9%. Only the pattern of Stagnated heat in liver-stomach performed third best with the accuracy 90.5% less than BP-MLL with the accuracy 91% and NL-kNN with the accuracy 90.8%. The perfect result demonstrates that the multilabel learning based on DBN method is superior to other multilabel learning methods.

## 6. Conclusions

To fully understand the characteristics of multilabel data of TCM in syndrome diagnosis, a deep learning model DBN is used to establish a multilabel learning framework and apply to TCM syndrome differentiation modeling for CG dates which are regarded as nonlinear and complicated. DBN based multilabel learning can perform outstanding for its capacity of high level information expression.

An experiment is set to find appropriate scale of nodes in one hidden layer DBN architecture with CG data. The result indicates that with only enough scale of nodes, but not too much, the DBN architecture can improve the performance of deep learning.

Moreover, DBN based multilabel learning was compared with other multilabel algorithms. Compared results indicated that DBN dealing with multilabel task performs better than other algorithms. The results are measured by five evaluation indexes; that is, average precision, coverage, hamming loss, one-error, and ranking loss. And all the indexes of DBN based multilabel learning achieve the best.

The study has shown that DBN based on multilabel learning is effective to deal with the task of modeling of TCM dates. In addition, the study will serve as a reference for establishing diagnostic criteria and a diagnostic model for CG and a better guide for clinical practice.

## Figures and Tables

**Table 1:** Results of model with different nodes' number (mean ± std.).

hid	Average precision ↑	Coverage ↓	Hamming loss ↓	One-error ↓	Ranking loss ↓
5	0.791 ± 0.019	0.191 ± 0.013	0.154 ± 0.011	0.304 ± 0.029	0.151 ± 0.016
10	0.802 ± 0.025	0.175 ± 0.019	0.144 ± 0.014	0.301 ± 0.043	0.133 ± 0.021
20	0.802 ± 0.029	0.175 ± 0.022	0.145 ± 0.012	0.303 ± 0.041	0.133 ± 0.026
30	0.817 ± 0.027	0.164 ± 0.021	0.139 ± 0.012	0.283 ± 0.039	0.120 ± 0.023
40	0.812 ± 0.017	0.168 ± 0.016	0.138 ± 0.015	0.292 ± 0.029	0.125 ± 0.017
50	0.815 ± 0.019	0.166 ± 0.018	0.137 ± 0.010	0.289 ± 0.027	0.123 ± 0.018
60	0.816 ± 0.023	0.164 ± 0.020	**0.135** ± **0.009**	0.287 ± 0.039	0.121 ± 0.020
70	0.818 ± 0.018	0.164 ± 0.015	0.139 ± 0.009	0.283 ± 0.028	0.120 ± 0.016
80	**0.823** ± **0.018**	**0.158** ± **0.015**	0.139 ± 0.014	**0.278** ± **0.028**	**0.116** ± **0.016**
90	0.821 ± 0.020	0.160 ± 0.016	0.137 ± 0.012	0.278 ± 0.034	0.116 ± 0.017
100	0.817 ± 0.023	0.166 ± 0.017	0.136 ± 0.006	0.281 ± 0.045	0.123 ± 0.018
200	0.818 ± 0.021	0.164 ± 0.016	0.139 ± 0.009	0.282 ± 0.035	0.120 ± 0.018
300	0.815 ± 0.023	0.164 ± 0.019	0.141 ± 0.011	0.287 ± 0.039	0.122 ± 0.022

**Table 2:** Results of model with different multilabel learning (mean ± std.).

	Average precision ↑	Coverage ↓	Hamming loss ↓	One-error ↓	Ranking loss ↓
ML-KNN	0.754 ± 0.031	0.206 ± 0.017	0.166 ± 0.017	0.380 ± 0.059	0.173 ± 0.020
BSVM	0.794 ± 0.037	0.180 ± 0.023	0.166 ± 0.022	0.320 ± 0.065	0.138 ± 0.029
Rank-SVM	0.682 ± 0.018	0.255 ± 0.029	0.232 ± 0.014	0.497 ± 0.025	0.227 ± 0.019
BP-MLL	0.514 ± 0.028	0.395 ± 0.036	0.313 ± 0.010	0.750 ± 0.048	0.390 ± 0.044
CLR	0.784 ± 0.024	0.185 ± 0.023	0.172 ± 0.016	0.343 ± 0.045	0.143 ± 0.021
ECC	0.793 ± 0.021	0.193 ± 0.018	0.150 ± 0.013	0.277 ± 0.038	0.193 ± 0.023
REKAL	0.781 ± 0.026	0.209 ± 0.024	0.152 ± 0.012	0.331 ± 0.036	0.167 ± 0.026
LEAD	0.803 ± 0.019	0.174 ± 0.016	0.151 ± 0.014	0.304 ± 0.034	0.133 ± 0.015
DBN	**0.823** ± **0.018**	**0.158** ± **0.015**	**0.139** ± **0.014**	**0.278** ± **0.028**	**0.116** ± **0.016**

**Table 3:** Results of recognition accuracy (%) for six common syndromes (mean ± std.).

Syndromes (patterns)	ML-kNN	BSVM	BP-MLL	Rank-SVM	DBN
Damp-heat accumulating in the spleen-stomach	86.9 ± 3.6	88.4 ± 2.5	24.7 ± 3.5	88.0 ± 2.8	**90.1** ± **0.024**
Dampness obstructing the spleen-stomach	73.7 ± 4.4	80.0 ± 3.5	68.3 ± 5.2	76.2 ± 4.4	**81.2** ± **0.019**
Spleen-stomach qi deficiency	68.9 ± 6.5	71.2 ± 2.3	53.8 ± 3.9	67.9 ± 6.8	**75.3** ± **0.044**
Spleen-stomach deficiency cold	96.6 ± 1.7	94.3 ± 2.7	96.6 ± 1.7	79.3 ± 3.6	**96.6** ± **0.021**
Liver stagnation	82.7 ± 5.6	82.6 ± 4.9	83.1 ± 5.4	81.0 ± 4.7	**83.9** ± **0.028**
Stagnated heat in liver-stomach	90.8 ± 2.3	90.1 ± 3.0	**91.0** ± **2.2**	79.9 ± 4.8	90.5 ± 0.030
